# Two novel temperate bacteriophages infecting *Streptococcus pyogenes*: Their genomes, morphology and stability

**DOI:** 10.1371/journal.pone.0205995

**Published:** 2018-10-19

**Authors:** Marek Harhala, Jakub Barylski, Kinga Humińska-Lisowska, Dorota Lecion, Jacek Wojciechowicz, Karolina Lahutta, Marta Kuś, Andrew M. Kropinski, Sylwia Nowak, Grzegorz Nowicki, Katarzyna Hodyra-Stefaniak, Krystyna Dąbrowska

**Affiliations:** 1 Bacteriophage Laboratory, Institute of Immunology and Experimental Therapy, Polish Academy of Sciences, Weigla 12, Wrocław, Poland; 2 Department of Molecular Virology, Faculty of Biology, Adam Mickiewicz University, Collegium Biologicum—Umultowska 89, Poznan, Poland; 3 DNA Research Center, Inflancka 25, Poznań, Poland; 4 Departments of Pathobiology; and, Food Science, University of Guelph, Guelph, Ontario, Canada; 5 Faculty of Biological Sciences, Wrocław University, Kuznicza 35, Wrocław, Poland; University of Helsinki, FINLAND

## Abstract

Only 3% of phage genomes in NCBI nucleotide database represent phages that are active against *Streptococcus* sp. With the aim to increase general awareness of phage diversity, we isolated two bacteriophages, Str01 and Str03, active against health-threatening Group A *Streptococcus* (GAS). Both phages are members of the *Siphoviridae*, but their analysis revealed that Str01 and Str03 do not belong to any known genus. We identified their structural proteins based on LC–ESI29 MS/MS and list their basic thermal stability and physico-chemical features including optimum pH. Annotated genomic sequences of the phages are deposited in GenBank (NCBI accession numbers KY349816 and KY363359, respectively).

## Introduction

Bacteria from the genus *Streptococcus* are well known to cause a serious healthcare burden. *Streptococcus pyogenes* can cause pharyngitis, rheumatic fever, glomerulonephritis, toxic shock syndrome (StrepTSS) and various skin infections including necrotizing fasciitis. Considered together these conditions may be responsible globally for ~500,000 deaths each year. Another important pathogen from the genus is *Streptococcus pneumoniae*. It is one of the leading causes of child morbidity and mortality worldwide by causing pneumonia, sinusitis, otitis, meningitis, bronchitis and febrile bacteremia [1–4]. On the other hand, members of the genus *Streptococcus* can be a normal part of the healthy human microbiome [1, 2, 5]. Streptococci form a significant part of the skin, oral and gastrointestinal microbiota and usually are harmless for the host. They may become pathogens, specifically in an immunodeficient host and/or by acquiring virulence factors that mediate pathogenicity of this bacterial group [5–8]. Some of the virulence factors that mediate the transition from a commensal to pathogenic strain are carried by temperate bacteriophages [6].

Due to the clinical significance of streptococci, viruses that infect these bacteria were investigated from the very beginning of studies on phages [9, 10]. Since then, a considerable array of bacteriophages infecting these bacteria has been isolated. Among the isolates, siphoviruses account for majority, but there are some podoviruses and very rare myoviruses [11–13]. Currently 3% of phage genomes in NCBI GenBank database are for *Streptococcus* phages. Slightly over 26% of them are classified to the *Siphoviridae* family, 10% are assigned to the *Podoviridae*, while the remaining 64% are unclassified [14]. Moreover, only 15 *Streptococcus* phages are classified to any genus approved by the International Committee on Taxonomy of Viruses (ICTV)—*Cp1virus*, *P68virus*, *Sap6virus*, *Sfi11virus*, and *Sfi21dt1virus* [15–17]. These genera soon will be renamed to *Cepunavirus*, *Rosenblumvirus*, *Saphexavirus*, *Brussowvirus* and *Moineauvirus*, respectively [16].

Here, we present data on two novel bacteriophages specific to *Streptococcus pyogenes*: vB_SpyS_Str01 and vB_SpyS_Str03. The basic physico-chemical characteristics of these phages were determined, their genomes sequenced and annotated; and, their structural proteomes identified by mass spectrometry.

## Results and discussion

### Isolation and basic characterization of two new temperate streptococcal phages

Bacteriophages were isolated from a clinical isolate of *S*. *pyogenes* (Polish Collection of Microorganisms at the Hirszfeld Institute of Immunology and Experimental Therapy, Polish Academy of Sciences (PCM) (PCM accession no. 2855), from plaques on agar plates after spontaneous release. Isolated phages were named Str01 and Str03, and were deposited in PCM (accession numbers 595-PH and 597-PH, respectively) and used for genomic, morphological and stability studies.

The phage host range was tested on a set of 37 clinical isolates of Group A *Streptococcus* (GAS) and 34 clinical isolates of Group B *Streptococcus* (GBS) (S3 Table). Str01 was active on 13.5% (n = 5) of GAS strains and 3% (n = 1) of GBS. Str03 phage was active on 51% (n = 19) of GAS and none of the GBS.

**Fig 1:**
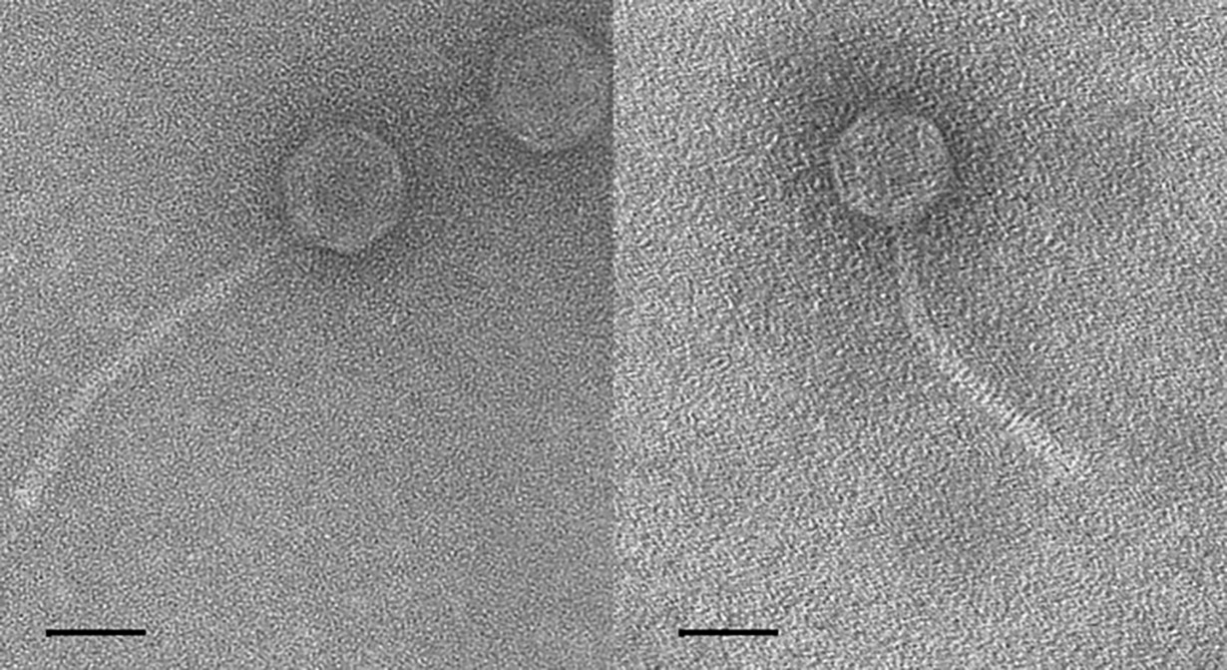
**Electron micrographs of phage Str01 (left) and Str03 (right).** Electron micrographs of negatively stained (2% uranyl acetate) phage Str01 (left) and Str03 (right). The scale bar represents 100 nm.

The morphology of these two phages was typical of siphoviruses and it is presented in the Fig 1 and the particle size was measured (S4 Table). Phage Str01 has a tail that is 186 nm long and a head which is 62 nm wide and 66 nm long. Phage Str03 has a tail that is 162 nm long and a head 62 × 66 nm.

The tolerance range of these two phages to pH and temperature was measured by incubation in BHI broth adjusted if necessary to selected pH and the results are shown in Tables 1 and 2.

**Table 1:** Stability of phages Str01 and Str03 in various temperatures.

Phage survivability (low temp.)							Phage survivability (high temp.)				
phage	str01			str03			phage	str01		str03	
temp	days			days			temp	minutes		minutes	
°C	5	40	180	5	40	180	°C	15	60	15	60
-80	102%	5.2%	4.9%	93%	33%	33%	40	98%	88%	100%	96%
-20	82%	2.3%	0.0%	66%	20%	0.0%	60	94%	0.0%	23%	1.0%
4	90%	11%	3.8%	85%	42%	12%					

**Table 2:** Stability of phages Str01 and Str03 in various pH values.

Phage survivability (pH)				
pH/time	Str01		Str03	
	1 h	5 h	1 h	5 h
3.0	0.0%	0.0%	0.0%	0.0%
3.5	0.0%	0.0%	0.0%	0.0%
4.0	0.2%	0.0%	0.1%	0.0%
4.5	29%	21%	78%	40%
5.0	63%	38%	101%	83%
7.0	92%	83%	99%	90%
9.0	42%	20%	46%	15%
11.0	30%	6.8%	29%	11%
11.5	21%	0.0%	27%	0.6%
12.0	0.0%	0.0%	0.0%	0.0%
13.0	0.0%	0.0%	0.0%	0.0%

Phage Str03 was more sensitive to freezing than Str01 (lower survival rate after five days in -20°C and -80°C), but is more stable in low temperatures during long term storage (survival rate 33% for Str03 versus 5% for Str01 at -80°C). Str03 shows higher sensitivity during incubation at high temperatures. After 15 min at 60°C 23% of Str03 phage particles remained biologically active in comparison to 96% of biologically active phage particles of Str01. Survival of both phages is similar in acidic and basic pH (incubations for 1 h and 5 h). Both phages lose biological activity after incubation in BHI broth with pH values lower than 4.0 and higher than 11.5.

Since both phages were isolated from PCM 2855 and the same strain is used as the host we were puzzled by the apparent lack of superinfection immunity. Two possible explanations for this situation are:

1. Phages were present in the sample collected from the environment and infected the PCM 2855 after isolation and deposition of this strain in PCM.
2. Str01 and Str03 are prophages in the genome of PCM 2855.

The second situation would have caused problems during phage growth and in other laboratory experiments. There would be a chance of contamination of Str01 samples with Str03 phage particles and *vice versa* by latent prophages from the host. We decided to test this hypothesis and prove whether PCM2855 contain prophages of Str01 and Str03 or not. We ran PCR with primers targeting phage specific sequences for both phages. We used lysogens from the host strain and used this as positive control (bacteria with phage-specific DNA). We have ensured that no free phage particles remained by washing bacterial cells prior to DNA extraction. This was validated by introduction of negative control (mix of *E*. *coli* cells and phage at concentration of 10^6^ PFU later washed using the same procedure prior to DNA extraction—see details in Materials and Methods).

**Fig 2:**
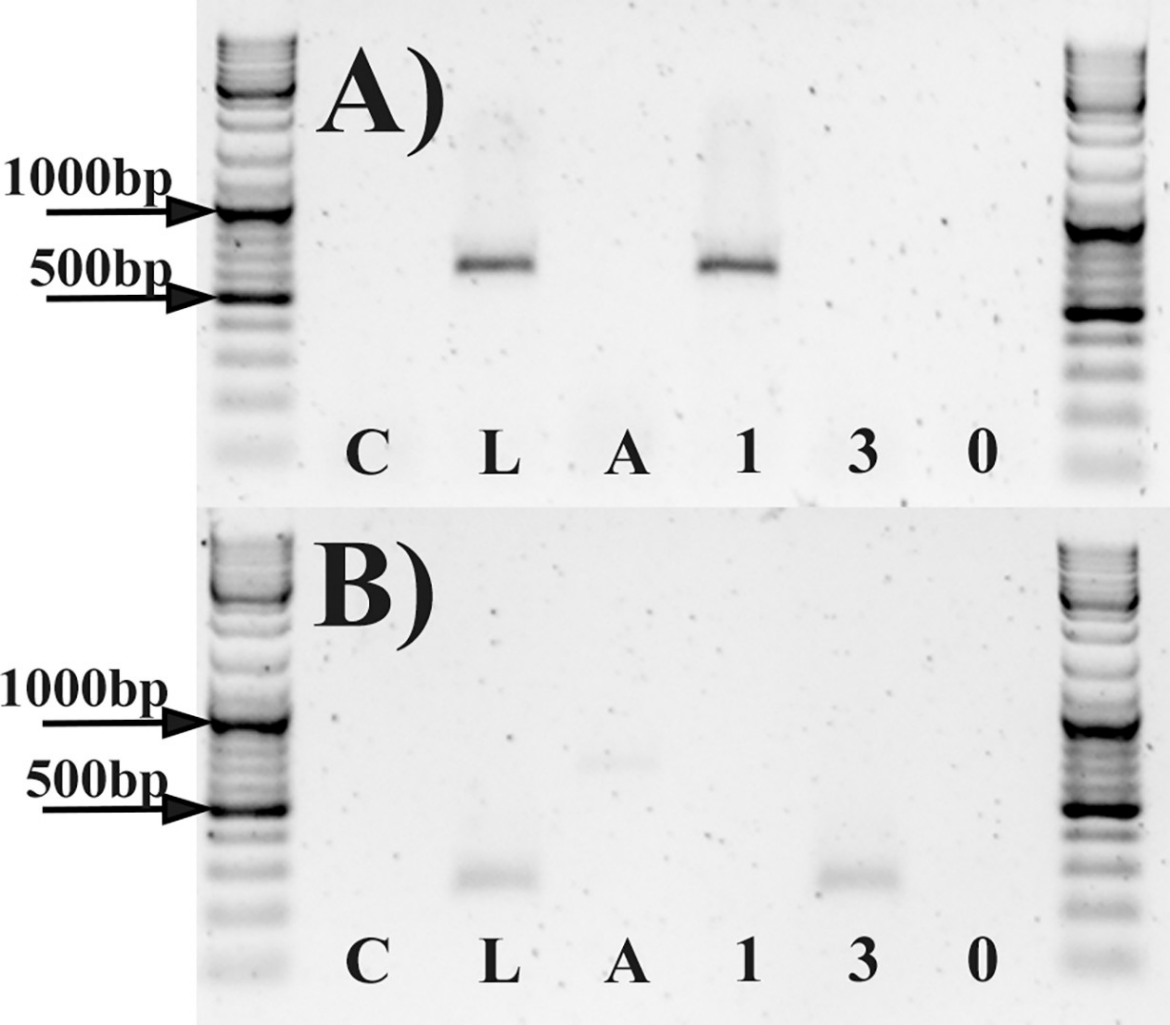
Result of agarose gel electrophoresis. (A) PCR with primers targeting sequence specific for Str01 genome. (B) PCR results with primers targeting sequence specific for Str03 genome. Each set of primers was tested with the same set of templates isolated from (left to right): C–*E*. *coli* and phage mix as wash protocol control; L–lysogenic strain of PCM 2855 (positive control), P–PCM 2855 host genome, 1 –phage genome Str01, 3 –phage genome Str03, 0 –no template (negative and environmental control).

We have confirmed that currently bacterial strain PCM 2855 is free of Str01 and Str03-like prophages. This conclusion is based mainly on the absence of Str01 and Str03 specific products after PCR reaction with host strain genome as template (Fig 2). Also there is no Str01 and Str03 genomes in the NGS raw data. This suggest that phages in the original sample were acquired from environment (by chance) and not as the phage present along bacterial host that later became deposited as PCM 2855.

We still cannot discard the second explanation completely. There is a chance that at some point during the course of isolation strain PCM 2855 either lost Str01 and Str03 or we isolated low-latent or immune to superinfection immunity versions of a phage already present as prophage. Such situation and possibilities were previously researched[18, 19].

### Genome structure and virion composition of Str01 and Str03 phages is typical for streptococcal siphoviruses but they carry some unusual genes

The phage genomes were sequenced with Illumina technology and each phage was assembled into a single gapless contig supported by read mapping and PCR verification. Their basic features are summarized in the Table 3 with more detailed information provided in S2 Table. The genomes of two phages are linear and may be circularly permuted since no software sequence assembly package could unambiguously delimitate their ends. Alternatively the assembly problem could be a result of Nextera library preparation method. It is known that this procedure can hinder end determination techniques based on statistical analysis of the read arrangement [20]. As a results the genome sequences were linearized to the presumed position of the beginning of the identified gene coding the small terminase subunit prior to annotation.

**Table 3:** Basic features of the analyzed genomes.

phage	genome size (bp)	GenBank Acc. no.	genes	GC (%)	Start codons (%)	Stop codons (%)	Coverage
Str01	37030	KY349816	48(+) 6(-)	37.9	ATG (98.1), GTG (1.9)	TAA (51.9), TGA (33.3), TAG (14.8)	906
Str03	32296	KY363359	43(+) 5(-)	39.1	ATG (91.7), TTG (6.3), GTG (2.1)	TAA (45.8), TGA (35.4), TAG (18.8)	2421

+/- symbols in “genes” column denote the strand of enumerated genes. Coverage values refer to the re-mapping of the reads to final PCR verified assembly (values reported by assembly software varied between 168.6 and 2261.7 depending on the tool used and the assembled library)

**Fig 3:**
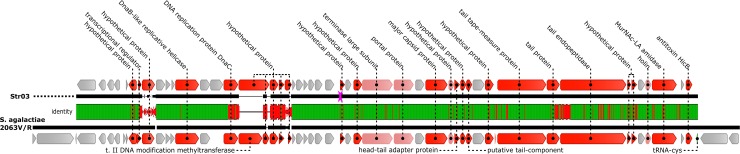
Similarities between genomes of Str03 and the most closely related phage. Genome maps are shown in a pairwise alignment. Arrows indicate genes and are colored according to the gene similarity (grey–identical genes, pink–genes with silent mutations, red–genes with amino acid substitutions in protein products). The middle bar shows DNA sequence similarity between the two genomes. Regions with no alignment are shown as a thin black line.

We found no phage genome with a significant (>70%) similarity to the Str03 but *Streptococcus agalactiae* strain 2603V/R (GenBank acc. no. AE009948) carries a very similar prophage element (91.6% pairwise identity) found between residues 558,765–599,345. This relationship is shown in Fig 3.

**Fig 4:**
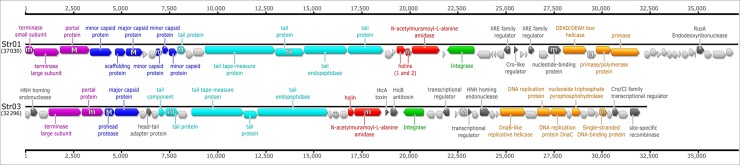
Genome organization of phages Str01 and Str03. Genome organization is shown as a linear map oriented to start at the small terminase subunit gene. Predicted CDSs are marked with arrows colored by predicted function: DNA packaging (violet); head assembly (blue), tail assembly (aquamarine), lysis (red), integration (green) and DNA replication (orange). CDSs with no function assigned are light grey. Genes with protein products detected by mass spectrometry are marked with the “M” or “m” symbol (depending on the confidence level of the finding see columns 6–8 in Table 4 and Table 5). The GC content of the isolated phages differs less than 1% from of the average of *S*. pyogenes and our phages follow typical features of such phages, like lack of phage-encoded tRNAs coding sequences. The genome maps were exported using Geneious software (v 9.0.5). Minor adjustments in the resulting svg file included realignment of labels (but not labelling itself) for the improved readability and addition of the “MS” symbols. All these adjustments were implemented using InkScape 0.91.

The general layout of the Str01 and Str03 genomes is shown in Fig 4 and follows the usual organization of siphoviruses with a clear modular organization and synteny recognizable even among distant relatives. One half of each genome encompasses clear-cut functional modules connected with DNA packaging, head and tail morphogenesis and lysis of the host cell. Module boundaries become less clear in the second part of both genomes. Nevertheless, regions involved in integration, DNA replication and lysogeny control are distinguishable.

**Fig 5:**
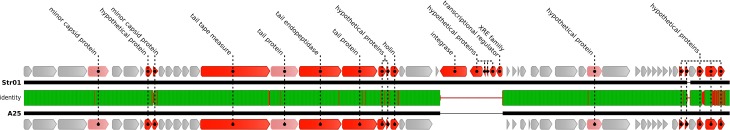
Similarities between genomes of Str01 and A25. Genome map of both phages are shown in a pairwise alignment. Arrows indicate genes and are colored according to the gene similarity (grey–identical genes, pink–genes with silent mutations, red–genes with amino acid substitutions in protein products). The middle bar shows DNA sequence similarity between the two genomes. Regions with no alignment are shown as a thin black line.

Phage Str01 encodes two independent holin genes. The first is a class I holin that encodes three transmembrane helices and possesses a holin 4_1 motif (pfam05105). The product of the second gene probably belongs to class III since only one transmembrane domain was identified at the end of the LL-H motif [18]. Similar arrangement was previously observed in case of *Streptococcus* phage A25 (Fig 5) which seems to be the closest known relative of Str01 (90.6% nucleotide identity). Interestingly, compared to the Str01 phage A25 seems to be missing ~3 regions carrying the integrase gene, XRE-family transcriptional regulator along with several unidentified genes.

**Fig 6:**
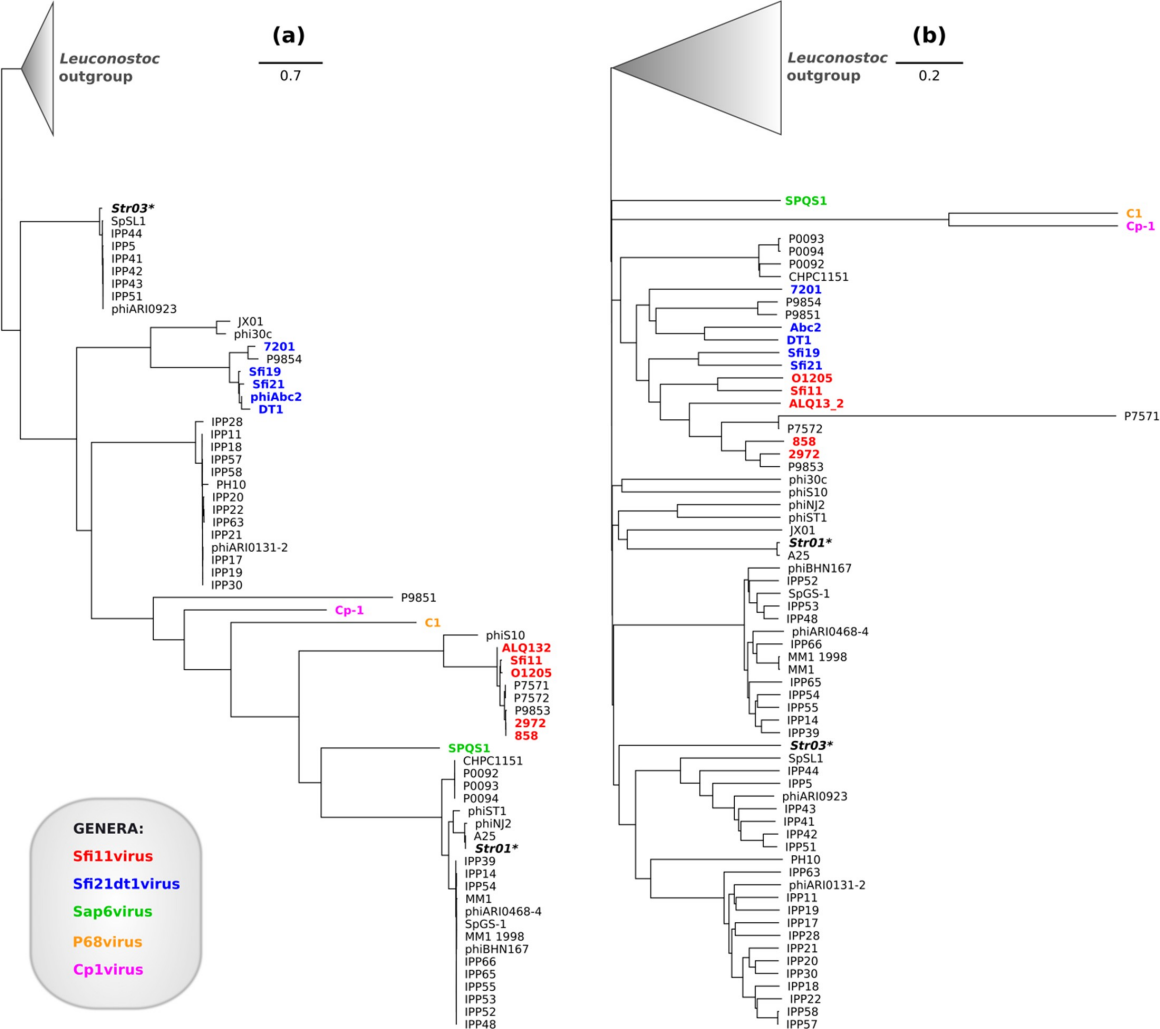
Phylogenetic trees showing relations between selected siphoviruses of Gram-positive bacteria. (A) Approximately maximum-likelihood tree based on alignment of large subunits of terminases. (B) Approximately Neighbor-joining tree based on whole genome comparison. The leaves are colored according to the ICTV classification of each phage. Names of phages Str01 and Str03 are italicized, bolded and marked with asterisk (*). The cone at the top of the tree represent collapsed branch of *Leuconostoc* phages treated collectively as the outgroup. All phylogenetic trees were visualized using Geneious tree viewer. Legend coloring and shading were added using InkScape 0.91 software.

Phage Str03 shows differences in comparison with phages with typical genome architecture. No gene encoding a small terminase subunit (TerS) was revealed by genome annotation. Thus, we closely examined the area adjacent to large terminase subunit where the *terS* gene is typically encoded. We considered that the gene might be disrupted by a mobile group I intron as this region is occupied by HNH homing nuclease [21]. Despite careful scrutiny, involving InterProScan, CD-Search and tBLASTn failed to reveal any trace of a TerS homologue While unusual, this situation is not entirely unique. Several groups of tailed phages do not encode any recognizable homologues of this protein. These includes streptococcal siphophages Dp-1, SpSl1 and phiARI0923 [22, 23], which possess large terminase subunit closely related to that of Str03 (Fig 6A). Full list of phages used for analysis is in S1 Table. In addition, genomes of *Lactococcus* phages, including c2, 4268, phiLC3 and Q54 [24] also lack any apparent TerS-coding gene. Thus, the lack of small terminase subunit in the Str03 genome may be a characteristic feature of the group of similar and possibly related bacteriophages. Interestingly, no phage sharing significant DNA-DNA similarity with Str03 over the whole genome length could be found. Instead we located a number of *Streptococcus agalactiae* prophages sharing very similar genome organization (and in case of most similar “LambdaSa2” prophage as much as 92.6% identity, see Fig 3 and S1 Table)

Another distinguishing feature of Str03 genome is the presence of a toxin-antitoxin cassette between the lysis and integration modules. The cassette is composed of a homologue of HicA mRNA interferase and a gene for its neutralizing factor–HicB. Similar elements were reported in streptococci but are rare in streptococcal phages [25, 26]. We propose that this cassette may be involved in forcing the host into the genetic addiction to the lysogenic state. Antitoxins typically degrade faster than toxins so the host doesn’t survive loss of the prophage [27, 28]. For additional information about similarity of HicA and HicB to other known proteins see S1 and S2 Figs, and S1 File.

### Phage structural proteins

We identified major components of the virion for both phages. Despite the CsCl gradient purification we detected contamination with some host proteins and non-structural phage proteins. Thus, we imposed stringent ion expect and protein p-value cutoffs to filter out contaminating proteins. The high scoring peptides supported the presence of main capsid proteins, portal proteins and a few tail components in the purified sample. Protein hits scoring beneath the threshold are presented in the Table 4 and Table 5 labeled “Low abundance proteins”. We propose that selected proteins (description in brackets) are phage structural proteins: APZ81889 (tail protein); APZ81895 (major capsid protein); APZ81902 (tail protein); APZ81903 (hypothetical protein); APZ81888 (portal protein); APZ81886 (tail tape measure protein); APZ81875 (minor capsid protein); APZ81901 (terminal small subunit); APZ81898 (nucleotide binding protein); APZ81883 (minor capsid protein) for Str01 and APZ82143 (hypothetical protein); APZ82134 (major capsid protein); APZ82138 (tail protein); APZ82157 (hypothetical protein); APZ82135 (portal protein); APZ82143 (hypothetical protein); for Str03. Mascot also reported the abundance of host membrane protein enolase in all samples of phage protein. This suggests that enolase, which is a surface-exposed adhesion protein of *Streptococcus* [29], may be a phage receptor or it may interact with proteins of studied phages in another way. This should be verified experimentally by the future studies.

**Table 4:** Proteins of the phage Str01 detected by mass spectrometry.

	no	NCBI Identical Protein Group	phage protein accession	phage protein defintion	score	% coverage	emPAI
A	1	23526059	APZ81895	major capsid protein	7931	78.1	6.35
	2	92043013	APZ81902	tail protein	410	34.2	3.37
	3	5668030	APZ81903	hypothetical protein	184	55.3	3.1
	4	23526069	APZ81888	portal protein	769	39.0	1.7
	5	92043017	APZ81889	tail protein	519	34.0	1.45
B	6	137081518	APZ81883	minor capsid protein	113	35.2	0.98
	7	23526073	APZ81882	holin	150	43.2	0.94
	8	14467	APZ81898	nucleotide-binding protein	135	22.1	0.91
	9	23526076	APZ81901	terminase small subunit	139	18.2	0.58
	10	43836092	APZ81891	N-acetylmuramoyl-L-alanine amidase	134	10.8	0.39
	11	23526079	APZ81875	minor capsid protein	112	6.8	0.21
	12	137081495	APZ81886	tail tape measure protein	183	4.9	0.15

Proteins are divided into ‘A’—“high abundance” and ‘B’—“low abundance” groups based on emPAI (Exponentially Modified Protein Abundance Index) parameter. Hit proteins were filtered based on: ions significance threshold (< 1e-5), ions expect cut-off (< 1e-5), overall protein score (> = 100) and emPAI (> = 0.15).

**Table 5:** Proteins of the phage Str03 detected by mass spectrometry.

	no	NCBI identical protein group	phage protein accesion	phage protein definition	score	% coverage	emPAI
A	1	33402471	APZ82134	major capsid protein	572	49.5	4.12
	2	103367874	APZ82143	hypothetical protein	165	37.0	1.39
B	3	354622	APZ82138	tail protein	282	30.0	0.95
	4	33235181	APZ82157	hypothetical protein	144	22.3	0.86
	5	1244846	APZ82147	single-stranded DNA-binding protein	114	20.3	0.69
	6	1184514	APZ82135	portal protein	252	15.8	0.47
	7	33302180	APZ82132	N-acetylmuramoyl-L-alanine amidase	130	5.8	0.18

Proteins are divided into ‘A’—“high abundance” and ‘B’—“low abundance” groups based on emPAI (Exponentially Modified Protein Abundance Index) parameter. Hit proteins were filtered based on: ions significance threshold (< 1e-5), ions expect cut-off (< 1e-5), overall protein score (> = 100) and emPAI (> = 0.15).

### Str01 and Str03 phages cannot be assigned to any known genus

No systematic, comprehensive and comparative analysis that definitely solves the conundrum of taxonomy of streptococcal phages has been published to date. This is likely the result of extensive gene transfer that blurs the division between the lineages and hinders the attempts to classify them. Perhaps, with the growing number of the sequences, clusters will finally emerge.

In an attempt to classify our phages we compiled the database of similar viruses. Then, we constructed two phylogenomic trees based on comparisons of whole genomes and terminase proteins of these viruses (Fig 6). On the resulting trees Str01 and Str03 failed to group with phages classified to any known genus. The sequence of Str01 phage is however similar to the genome of *Streptococcus* phage A25 which is classified as a member of the *Podoviridae*. The Str03 terminase is identical to the of prophage LambdaSa in a few *Streptococcus agalactiae* strains. This protein is also 95% identical to TerL from phages IPP5 (KY065449), IPP41 (KY065481), IPP42 (KY065482), IPP43 (KY065483), IPP44 (KY065484), IPP51 (KY065489), phiARI0923 (KT337370) and SpSL1 (KM882824) while the rest of the genome is not. All of these phages remain unclassified and their relations with other streptococcal phages are uncertain. We propose that the inconsistency results from the horizontal gene transfer that impairs clear demarcation. Despite this, the topology of the resulting trees roughly conforms to current taxonomic scheme and clusters corresponding to known genera are recognizable. Only the *Sfi21dt1virus* (*Moineauvirus*) genus appears polyphyletic in the whole genome tree. To sum up, Str01 and Str03 cannot be fitted into any genus; they are rather representatives of separate lineages. Perhaps Str01 and Str03 phages will eventually be included into newly delineated genera but this fall outside the scope of this study.

## Materials and methods

### Isolation of new phages

Phage isolation was conducted using a liquid culture of *Streptococcus pyogenes* strain from Polish Collection of Microorganisms at Hirszfeld Institute of Immunology and Experimental Therapy, Polish Academy of Sciences (PCM) was used (PCM accession no. 2855). After 18 h incubation in BHI broth at 37°C bacterial cultures were centrifuged (8,000 x g, 5 min) and filtered through sterile filters (pore size: 0.2 μm). Filtrates were used in a plaque assay on double-layer BHI plates with the same *Streptococcus pyogenes* strain. Transparent plaques were selected and reisolated from single plaques for five times. Samples were named Str01 and Str03 and they were deposited in PCM (PCM accession no. 595-PH and no. 597-PH, respectively). Their host-range was assessed by spot-test on 33 *S*. *pyogenes* and 34 *S*. *agalactiae* strains cultured on BHI plates.

### Analysis of physico-chemical properties

Phage lysates were incubated (from 0°C to 60°C) or frozen (-80°C and -20°C) in BHI broth at different temperatures to test thermal resistance of Str01 and Str03 and imitate conditions of phage storage. Also phage samples were incubated in BHI broth at different pH values (from pH = 3.0 to pH = 13.0) for 1 h and 5 h at 37°C. The concentration of phages was measured by dilution method (BHI plates) and compared with the concentration of the sample on the beginning. Each incubation was performed twice with three independent samples.

### DNA isolation

Phage lysate (at least 10^10^ PFU) was concentrated by addition of NaCl (final concentration of 0.5M) and PEG 8000 (final concentration of 10% w/v) (Sigma-Aldrich). After incubation at 0°C for 16 h overnight, the samples were centrifuged (15,000 x g, 15 min, 4°C) and the pellet was resuspended carefully in TE buffer for 3 h. MgCl_2_, RNase and DNase (Omega Bio-tek) were added to 0.5 mM, 60μg/ml and 30 μg/ml, respectively and incubated for 30 min at 37°C). Proteinase K (100 μg/ml) was added and the incubated was continued for an additional 10 min. The samples were purified GenElute Mammalian Genomic DNA Miniprep Kit (Sigma-Aldrich) following to manufacturer’s protocols. The DNA was precipitated with ethanol, the pellet washed with precooled 75% ethanol, and finally air dried. The samples was resuspended in DNase, RNase free H_2_0.

### Sequencing and annotation of genomes of the *S*. *pyogenes* phages

The DNA sample quantity and the purity of the nucleic acid samples were assessed using a Nanodrop spectrophotometer (Thermo Fisher Scientific) and agarose gel electrophoresis. Prior to library preparation the concentration of the isolated DNA was rechecked using Qubit dsDNA HS kit (Invitrogen, Life Technologies). Libraries were constructed using the Illumina NexteraXT DNA Library Prep Kit. 5μl of normalized DNA (0.2 ng/μl per sample) was used for the tagmentation reaction, which is a process that fragments DNA and simultaneously adds adapter sequences to the DNA, compatible with Illumina’s indices. PCR added indices containing P5/P7 adapters to make the library compatible with the flow cell. All reactions were set up in duplicate and incubated as per the manufacturer’s instructions. Reactions were cleaned up using AMPure XP beads (Beckman Coulter) in a concentration of 0.6X AMPureXP Beads. Reactions were eluted with RSB (Illumina Inc., San Diego, CA, USA). Finally, size assessment was performed using Experion Bioanalyzer (BioRad). Concentrated of amplified DNA fragments were normalized to 2nM using Qubit dsDNA HS kit (high sensitivity DNA; Life Technologies). 10 pM library pool, consisting of pooled indexed samples, was loaded on the MiSeq platform (Illumina). 150-nucleotide-long paired-end sequencing run was performed on the MiSeq with addition of 10% spiked-in ΦX-174 control DNA.

Reads were trimmed using Trimmomatic [30] and their quality was assessed with FastQC version 0.11.3 [30] Then genomes were assembled using MIRA 4.9.5_2 [31] SPades 3.1 [32] and Geneious 9.0.5 [33]. Independent assemblies were compared and their quality was assessed by mapping reads back to each of them with Geneious mapping algorithm. Uncertain or ambiguous regions were resolved by inspection of the read mapping and, if needed, by PCR amplification and Sanger sequencing.

Protein coding genes were predicted using GeneMarkS, GeneMark.hmm [34], Glimmer 3 [35], RAST [36], FGENESV (Softberry, Inc.) and Prodigal 1.20 [37]. CDSs with no overlapping BLASTx hits (against the NCBI nr database) predicted by only a single tool were discarded. Conflicting start codons were resolved based on RBS positions (predicted by Prodigal and by scanning for motifs overrepresented in regions 1–20 nt upstream predicted genes with MEME suite) as well as BLAST alignments [38]. BLASTx analysis was also used for functional annotation of CDSs. Predicted coding sequences were then translated and their initial annotation was manually re-assessed using BLASTp, InterProScan 5 and CD-Search [39–41]. tRNA genes were predicted by tRNAscan-SE version 1.21 [42].

As no apparent physical termini could be found by examination of the read arrangement (either manual or with PAUSE software) annotated genomes have been linearized to start with the small terminase subunit (or in case of the Str03 in its expected position, conventional starting point of *Siphoviridae* genomes).

### Phylogenetic analysis

To gain insight into the evolutionary history of Str01 and Str03 and determine their taxonomic position we studied both their whole genomes and terminase proteins.

First, we compiled a set of similar genomes for phylogenetic analysis based on the results of the BLAST analysis. Beside studied phages we included all non-redundant viral genomes from nr/nt database that reached e-value < 1e-25 in either BLASTn search with both genomes as a query or tBLASTn search with Str01 and Str03 terminases as a protein query.

After the curation of a compiled set we performed whole genome comparison of all included phages using Gegenees 2.2.1 with used “accurate” BLASTn settings (the tool global similarity between pairs of sequences based on BLAST local alignments). The resulting similarity matrix was used to construct Neighbor Joining phylograms with SplitsTree 4.14.1 (www.splitstree.org/). The BLAST search, CD-Search and manual curation was required to locate all terminase genes (S1 and S2 Tables). We aligned them using ClustalW (default parameters) and refined the alignments using MUSCLE. Suitability of protein evolution models was assessed with ProtTest 3.4 [27]. The chosen model (WAG+G) was used to calculate approximately maximum-likelihood dendrogram with FastTree 2.1.7 [28].

### Analysis of the phage proteins

Remains of unlysed bacteria were removed from the lysates by centrifugation and the phage was concentrated by PEG precipitation (0.5 M NaCl and 10% PEG). After incubation on ice overnight (18 h) samples were centrifuged (15,000 × g, 15 min, 4°C). Supernatant was discarded and the pellets resuspended in TE buffer. Any undissolved particulates were removed by centrifugation (3000 × g, 10 min, 4°C). The supernatant was dialyzed into SM buffer (100 mM NaCl, 10 mM MgSO4, 50 mM Tris-HCl, pH 7.5). Cesium chloride (0.75 g/ml) was added to prepare the sample for density gradient centrifugation. The mixture was centrifuged for 24 h in 155,000 × g at 4°C. Phage-containing band that formed was carefully collected and dialyzed at for 24 h against SM buffer supplemented with 1 M NaCl (final concentration). After additional round of dialysis against standard SM buffer (3 h in room temperature) phage suspension was filtered-sterilized (0.22 μm pore size; Millipore). Sterilized sample was mixed with methanol and chloroform (1:1:0.75 by volume). The aqueous phase was separated from organic solvents by centrifugation and discarded. Equal volume of methanol was added to the remaining organic fraction and interface precipitate, then protein was collected by centrifugation at 21,500 × g at 4°C. The pellet was dried, suspended in Laemmli sample buffer (4% SDS, 20% glycerol, 10% 2-mercaptoethanol, 0.004% bromophenol blue and 0.125 M Tris.HCl, pH 6.8) and resolved on 12% polyacrylamide gel.

Then, resolved proteins were analyzed by LC–ESI-MS/MS analysis conducted in Mass Spectrometry Laboratory, Institute of Biochemistry and Biophysics, PAS, Warsaw, Poland. The whole lane was treated with trypsin and released peptide mixture was analyzed using Thermo Orbitrap Elite coupled with Thermo EASY-nLC 1000.

### Lysogen isolation and prophage testing (PCR)

Lysogens were obtained by pouring increasing 10-fold dilution of the phage onto the lawn of the host bacteria. After one day we checked for clear (transparent) plaques and after another two days we checked for opaque bacteria lawn inside of previously transparent plaques (so called 'mesa'). We streak the cells onto fresh plates and isolated 3 colonies after 24h incubation. We streak them again and isolated two other colonies from each sample.

Wash protocol was introduced in order to remove any present free-floating phage particles. Bacteria (designed for template in PCR) were harvested and washed by suspending cells in PBS (50ml) and unwanted debris were removed by centrifugation (8000g, 5min, 4°C). This wash step was repeated 8 consecutive times to remove any free phage particles from the sample. Bacterial template was obtained by heating the sample resuspended in PBS at 99°C for 10min and further 100 fold dilution. This wash protocol was evaluated by introduction of a negative control that consist of DNA isolated from specially prepared sample. 10ml of overnight culture of *E*. *coli* BL21 (as non-host strain) and the phage (10^6 CFU) were thoroughly mixed and washed as in mentioned above protocol.

PCR reaction consisted of two negative controls (0 –without added template and C–mix of *E*. *coli* and a phage as wash protocol negative control), positive control (L–lysogen strain of PCM 2855 and the phage) and—two specificity controls (1, 3—purified genomes of Str01 and Str03 respectively). Two set of primers were tested: one specific against Str01 phage (TGCGGACACTGACAAAATTTTTGG and GGGGGATAAAAATGAATGAAACGCT) and the second one specific against Str03 phage (TACTCTGATCATTGGCTTAATCTAAT and GCTTGGCAGTGTGACAGTCTTG).

## Supporting information

S1 Table Database records used in this study. Accession numbers of all phages genomes and terminase proteins used in Fig 6.
(XLSX)

S2 Table Genes of novel *S*. *pyogenes* phages. First two sheets sum up predicted CDSs, their protein products and data used during their annotation (BLAST hits and InterProScan domains). The third tab shows the information on putative Shine-Dalgarno sequences found upstream the genes. Models of Shine-Dalgarno sites were calculated using the MEME (as a most significantly overrepresented 6–8 bp long motifs in regions 4–16 bp upstream of the each phage genes). Occurrences of these motif were located using FIMO. The workbook contains annotated DNA and protein sequences in gff and GenBank format (embedded in the relevant tabs of the xmlx file).
(XLSX)

S3 Table Host range test results (+ turbid plaques, ++ almost opaque plaques, +++ opaque plaques). Results of host range experiment. Lysis was checked after incubation of bacterial strain with bacteriophage on agar plates. Susceptibility was determined in comparison of samples with agar plate with resistant bacterial strain and plate with phage host without added phage.
(XLSX)

S4 Table Results of bacteriophage particle size measurements. EM photographs with scale bars were measured. Only undamaged virion particles filled with DNA were measured.
(XLSX)

S1 Fig Approximate maximum likelihood tree of HicA proteins related to Str03 HicA toxin. The Str03 homologue is marked with the red arrow. Sequences were aligned using ClustalW plugin from the Geneious suite, the FastTree plugin was used to construct the tree and Geneious Tree Viewer was used to export the figure. To improve the readability we visualized only the subtree representing a major branch including Str03 sequence.
(PDF)

S2 Fig Approximate maximum likelihood tree of HicB proteins related to Str03 HicA antitoxin. The Str03 homologue is marked with the red arrow. Sequences were aligned using ClustalW plugin from the Geneious suite, the FastTree plugin was used to construct the tree and Geneious Tree Viewer was used to export the figure. To improve the readability we visualized only the subtree representing a major branch including Str03 sequence.
(PDF)

S1 File Zip archive with complete tree files used to generate S1 and S2 Figs (in PHYLIP format). (PHY)
