# Supplementary figures and images for: Two novel temperate bacteriophages infecting Streptococcus pyogenes: Their genomes, morphology and stability
Source: PLoS One. 2018 Oct 19;13(10):e0205995. doi: 10.1371/journal.pone.0205995 (PMC6195288; doi:10.1371/journal.pone.0205995)

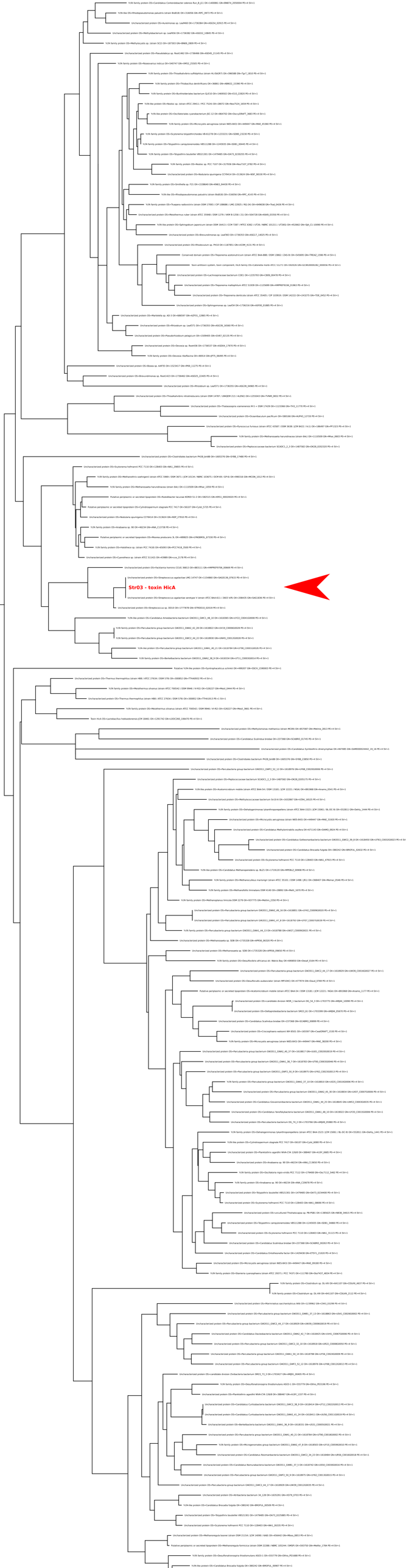

Supplement: S1 Fig — The Str03 homologue is marked with the red arrow. Sequences were aligned using ClustalW plugin from the Geneious suite, the FastTree plugin was used to construct the tree and Geneious Tree Viewer was used to export the figure. To improve the readability we visualized only the subtree representing a major branch including Str03 sequence. (PDF) [file pone.0205995.s005.pdf]

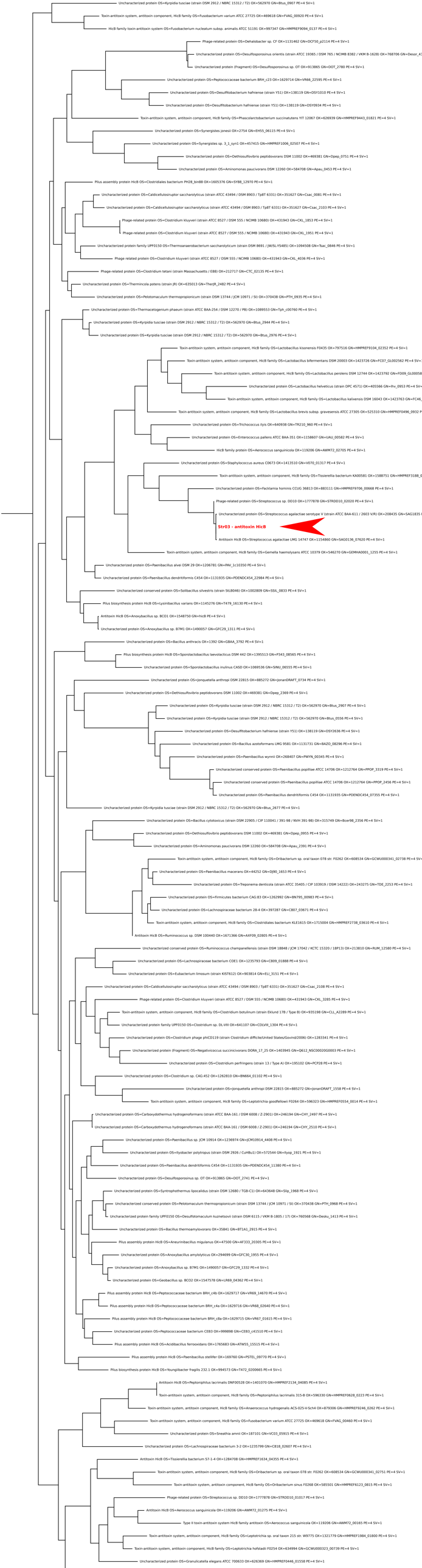

Supplement: S2 Fig — The Str03 homologue is marked with the red arrow. Sequences were aligned using ClustalW plugin from the Geneious suite, the FastTree plugin was used to construct the tree and Geneious Tree Viewer was used to export the figure. To improve the readability we visualized only the subtree representing a major branch including Str03 sequence. (PDF) [file pone.0205995.s006.pdf]
